# Supplementary material for: Establishing age and gender-specific serum creatinine reference ranges for Thai pediatric population
Source: PLoS One. 2024 Mar 12;19(3):e0300369. doi: 10.1371/journal.pone.0300369 (PMC10931469; doi:10.1371/journal.pone.0300369)
Supplement: S1 File — (PDF) [file pone.0300369.s001.pdf]

Supplementary Information

Establishing Age and Gender-Specific Serum Creatinine Reference Ranges for Thai Pediatric Population: A Retrospective Analysis

Sakon Suwanrungroj[1], Parichart Pattarapanitchai[2], Sirinart Chomean[3, 4], Chollanot [3,4*]

[1]Queen Sirikit National Institute of Child Health, Thung Phayathai Subdistrict, Ratchathewi, Bangkok, 10400, Thailand

[2]Department of Statistics, Faculty of Science, Chiang Mai University, Chiang Mai 50200, Thailand

[3]Department of Medical Technology, Faculty of Allied Health Sciences, Thammasat University, Pathum Thani 12120, Thailand

[4]Thammasat University Research Unit in Medical Technology and Precision Medicine Innovation, Pathum Thani 12120, Thailand

*Corresponding author

Email: Chollanotk@gmail.com

These authors contributed equally to this work.

S1 Fig. Presents histograms of data for the age group 11-<13 years, females: (**A**) represents the raw data, (**B**) depicts data after outlier exclusion, and (**C**) shows data post outlier exclusion and adjustment using the Box-Cox transformation.

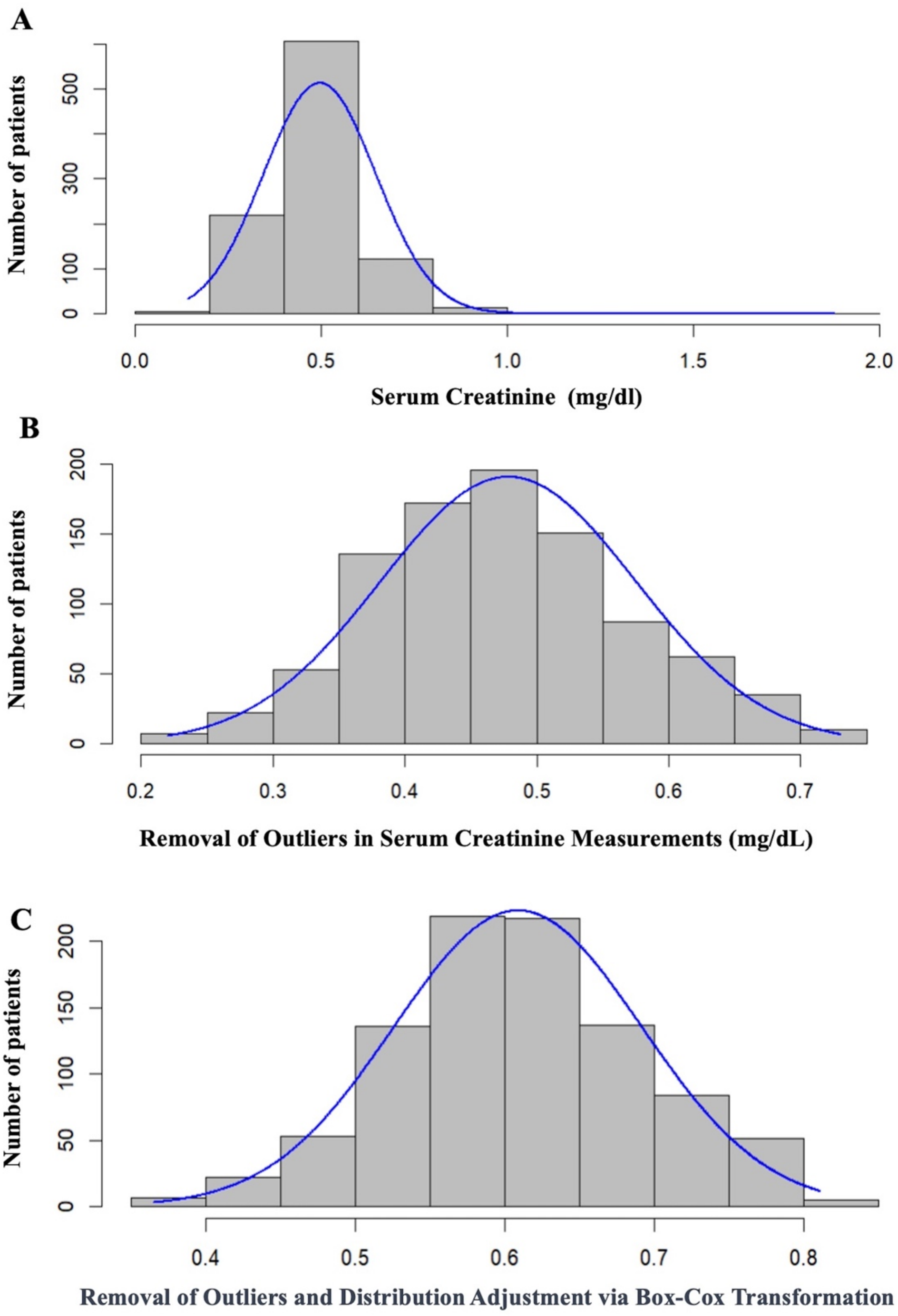

S2 Fig. Scatter plot illustrating the relationship between serum creatinine levels and age in the 0-60 days age range. (A) Male and (B) Female

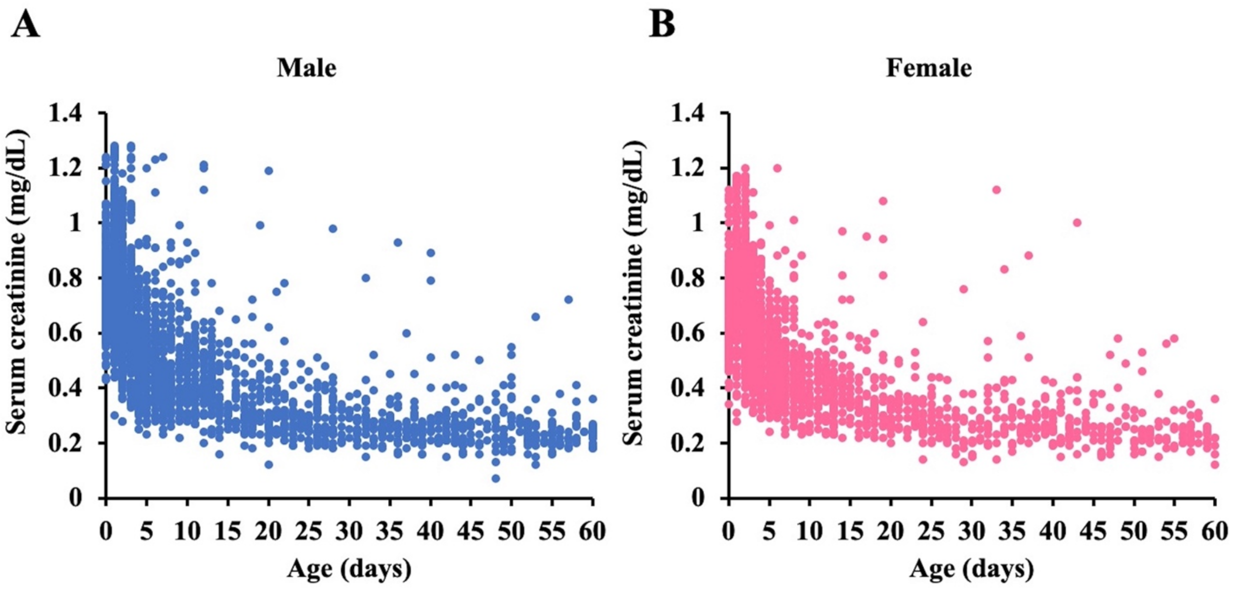

S3 Fig. Scatter plot demonstrating the relationship between serum creatinine levels and age ranging from 0 to 6,570 days, or 0 to 18 years. (A) Male and (B) Female

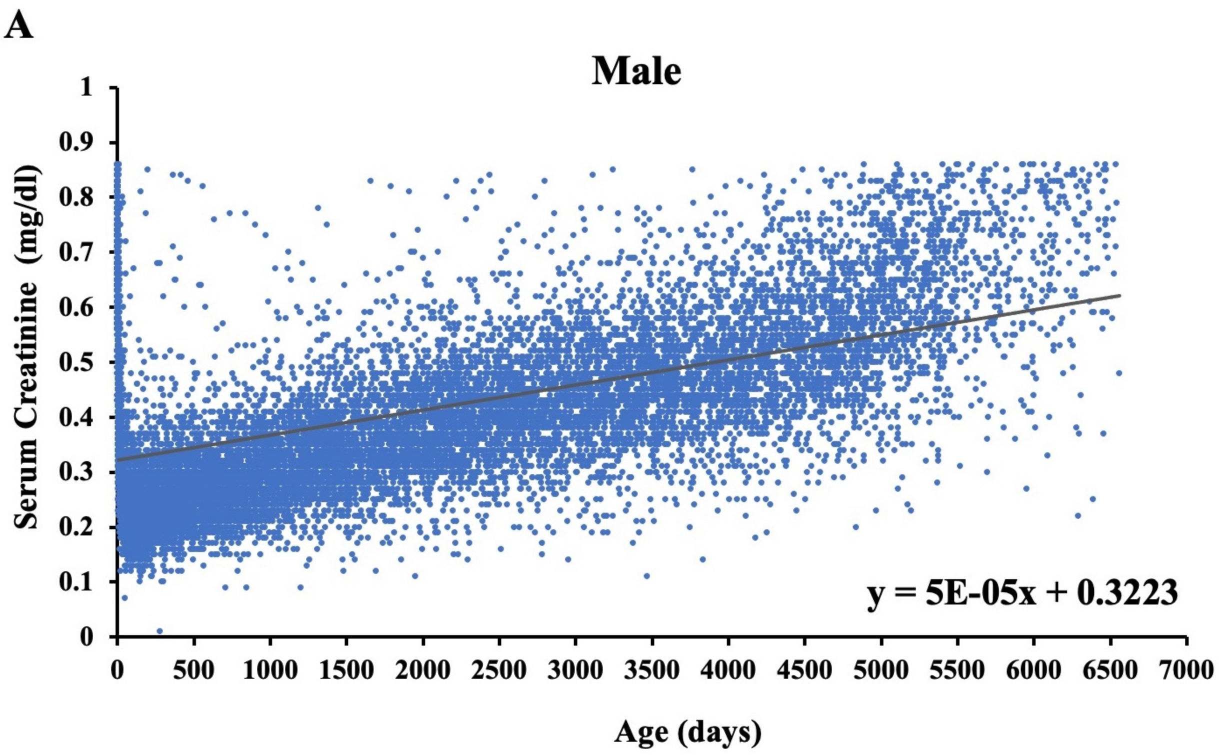

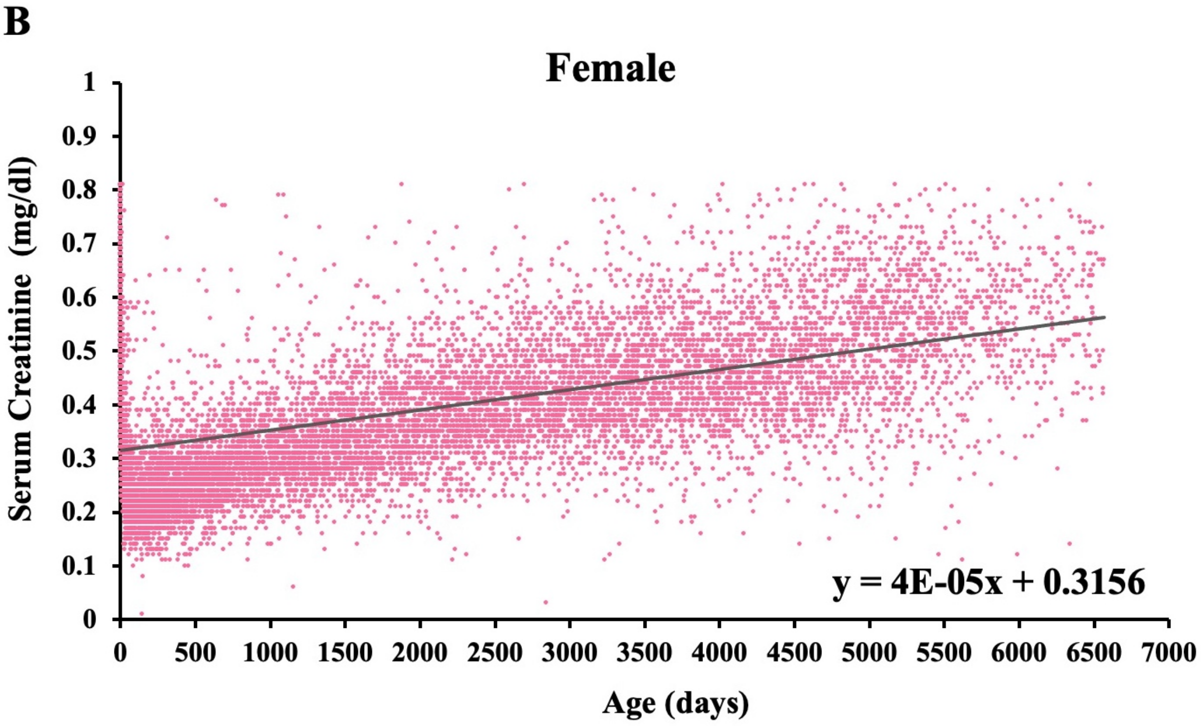

S4 Fig. Line graph illustrating the comparison of upper and lower limits of serum creatinine in both male and female subjects.

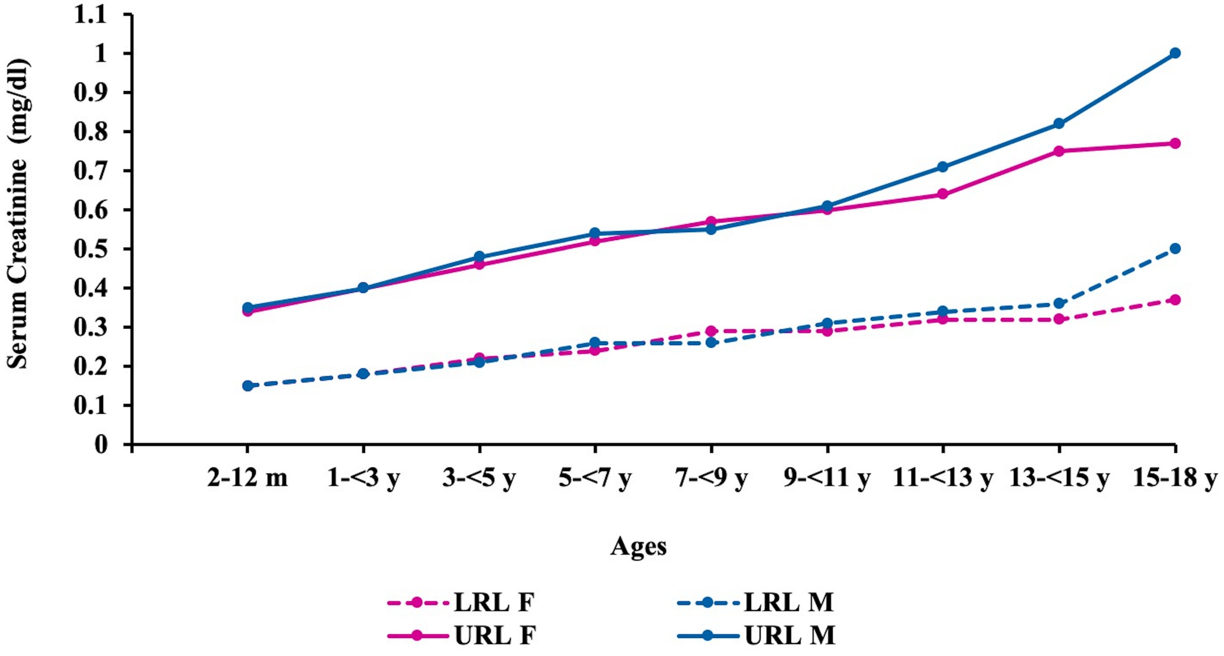

S1 Table. Presents the data for 20 age and gender groups after outlier exclusion. Includes sample size, mean, median, standard deviation, and coefficient of variation for each group

**Age**	**Sex**	**n**	**Mean**	**Median**	**SD**	**CV**
0-60 d	F	1851	0.53	0.50	0.23	43.40
	M	2578	0.55	0.53	0.24	43.64
2-12 m	F	1334	0.23	0.22	0.05	21.74
	M	1829	0.23	0.23	0.05	21.74
1-<3 y	F	1811	0.28	0.27	0.06	21.43
	M	2311	0.28	0.27	0.06	21.43
3-<5 y	F	1280	0.33	0.33	0.07	21.21
	M	1603	0.34	0.34	0.07	20.59
5-<7 y	F	1117	0.39	0.38	0.08	20.51
	M	1473	0.39	0.39	0.08	20.51
7-<9 y	F	1113	0.43	0.42	0.08	18.60
	M	1285	0.44	0.43	0.08	18.18
9-<11 y	F	1076	0.45	0.45	0.09	20.00
	M	1235	0.48	0.47	0.09	18.75
11-<13 y	F	931	0.48	0.47	0.10	20.83
	M	1155	0.52	0.52	0.10	19.23
13-<15 y	F	790	0.53	0.54	0.12	22.64
	M	943	0.62	0.61	0.14	22.58
15-18 y	F	488	0.57	0.56	0.11	19.30
	M	443	0.72	0.73	0.15	20.83

S2 Table. Displays the % difference in lower limits of the established reference values compared to the reference values from the studies of Schlebusch H et al., Pottel H et al., and Chuang GT et al.

Age	Sex	Our study	Schlebusch et al.		Pottel et al.		Chuang et al.	
		LRL	LRL	% Difference	LRL	% Difference	LRL	% Difference
2-12 m	F	0.15	0.16	-6.7	-	-	-	-
	M	0.15		-6.7				
1 - < 3 y	F	0.18	0.18	0.0	0.18	0.0	0.15	16.7
	M	0.18		0.0		0.0	0.15	16.7
3 - < 5 y	F	0.22	0.26	-18.2	0.23	-4.5	0.2	9.1
	M	0.21		-23.8		-9.5	0.21	0.0
5 - < 7 y	F	0.24	0.29	-20.8	0.25	-4.2	0.24	0.0
	M	0.26		-11.5		3.8	0.25	3.8
7 - < 9 y	F	0.29	0.34	-17.2	0.28	3.4	0.28	3.4
	M	0.26		-30.8		-7.7	0.28	-7.7
9 - < 11 y	F	0.29	0.33	-13.8	0.34	-17.2	0.31	-6.9
	M	0.31		-6.5		-9.7	0.33	-6.5
11 - < 13 y	F	0.32	0.44	-37.5	0.38	-18.8	0.33	-3.1
	M	0.34		-29.4		-11.8	0.36	-5.9
13 - < 15 y	F	0.32	0.46	-43.8	0.42	-31.3	0.42	-31.3
	M	0.36		-27.8		-16.7	0.42	-16.7
15 - 18 y	F	0.37	-	-	0.42	-13.5	0.42	-13.5
	M	0.5		-	0.47	6.0	0.55	-10.0
