# Supplementary material for: Establishing age and gender-specific serum creatinine reference ranges for Thai pediatric population
Source: PLoS One. 2024 Mar 12;19(3):e0300369. doi: 10.1371/journal.pone.0300369 (PMC10931469; doi:10.1371/journal.pone.0300369)
Supplement: Attachment — Submitted filename: Response to Reviewers .pdf [file pone.0300369.s004.pdf]

Thank you for dedicating your valuable time to reviewing our manuscript. Your thoughtful insights and constructive feedback are greatly appreciated. Below, we provide a point-by-point response to each of your comments:

Reviewer 1

Page 4, lines 82-87

1. Can the exclusion criteria be more stringent? What was the general health status of the subjects? If they were healthy, how do you define or verify that?

Response 1: Thank you for your insightful comments and queries regarding our study. We appreciate your attention to detail and the opportunity to address your concerns. This study utilizes a database of children's information, which has been compiled on a large-scale hospital database. The criteria for inclusion were set based on the desired study period to ensure sufficient data and a sample group that is current and closely resembles the population for which the established reference values will be used. Due to limitations in the number of children's data, setting inclusion criteria by selecting only clinics for healthy children, which normally do not collect specimen samples or have very few data, may result in insufficient data and the need to collect data over many decades, making the sample group not current. Changes in the population over time is another factor that could cause the reference values to change. In our study, the sample group used must represent healthy children or children without diseases (where the diseases are not related to changes in creatinine levels, such as ophthalmology clinics, brain diseases, etc.) Exclusion criteria were used to select a representative sample of healthy children, derived from collecting exclusion criteria from previous studies on creatinine reference values and considering creatinine as a marker for screening kidney function efficiency. Hence, children who are healthy and visit the hospital are also screened for creatinine as a preliminary test to help screen for abnormalities. Therefore, the established exclusion criteria are sufficiently effective to select out data of children with diseases, leaving

only data that represent healthy individuals for the study. Furthermore, in this reference value creation process, the research team used statistical methods to eliminate such abnormal data, using the Tukey test for removing outliers, where data with abnormalities are excluded in this step. Additionally, the creation of reference values using the Bhattacharya method is recommended by the IFCC as an efficient method that can distinguish data between normal individuals and those with diseases. Thus, statistical methods and the Bhattacharya method help eliminate such data from the dataset.

Page 4, lines 65-57

2. How about conditions that could have indirectly impacted on renal function e.g. severe dehydration?

Response 2: Thank you for highlighting this aspect, and we appreciate the opportunity to further refine our approach to ensure the robustness and accuracy of our findings.

Some indirect effects that are conditions of the body that may arise from diseases affecting the kidneys and influencing serum creatinine levels can occur in some patients. Access to individual patient data, which is necessary for this aspect, presents a limitation in accessing patient information, including the large volume of data that cannot be screened for these abnormalities in every patient case. Therefore, it is not possible to use certain conditions that occur in some individuals as exclusion criteria. As a result, some conditions causing abnormal creatinine levels may be included in the dataset. However, in this reference value creation process, the research team has used statistical methods to eliminate such abnormal data, through the removal of outliers using the Tukey test, where data with abnormalities are excluded at this stage. Additionally, the creation of reference values using the Bhattacharya method is also recommended by the IFCC as an efficient method that can distinguish data

between normal individuals and those with diseases. Thus, statistical methods and the Bhattacharya method help eliminate such data from the dataset. Moving forward, we will explore opportunities to incorporate measures or controls for hydration status in our study protocols to better account for potential indirect impacts on renal function. This may involve collecting additional data on hydration status or implementing measures to standardize hydration levels across study participants.

Page 5, lines 99-104

3. Good to see QC activities to reduce analytical errors. What considerations were made for the minimization of the inclusion of results associated with pre-analytical errors?

Response 3: Thank you for your insightful comment , Every step of operation from pre-analytical, analytical, and post-analytical phases in laboratory practice has been certified for quality by the Laboratory Accreditation (LA) using the ISO/FDIS 15189 standard of the year 2000 as the foundational basis, and the Health Accreditation (HA), which is certified by an international organization, The International Society for Quality in Health Care External Evaluation Association (ISQua EEA). The pre-analytical process at every stage must be standardized and verifiable, including 1. The expertise of personnel, who must undergo training according to professional standards and operate in a uniform manner, as demonstrated in their professional records. 2. Laboratory equipment must be ready for use, calibrated, and maintained according to requirements, with documentation to demonstrate the maintenance plan and quality of every piece of equipment. 3. Control of the process in the pre-analytical phase, with quality control documents and laboratory manuals, starting from 3.1 sample storage, such as patient preparation, 3.2 methods of sample collection, such as type and volume of samples, etc., 3.3 sample transportation, reception, and storage, ensuring that every pre-

analytical step complies with professional standards and hospital standards, which are internationally accredited. The revised sentence has been added to the manuscript on **Page 5, Lines 105-110, and Page 6, Lines 111-114, respectively**

4. Page 10, lines 197-198
Results on age group (newborn - < 2months were not presented

Response 4: Thank you for your valuable feedback and for taking the time to review our manuscript. Apologies for the oversight, and thank you for bringing it to our attention. We have now included the results for the age group (newborn - < 2 months) in **Table 1**. Additionally, a revised explanation of Table 1 has been incorporated into the revised manuscript on **pages 9-10, lines 192-206**. This table illustrates the upper and lower limits of serum creatinine reference values (in mg/dL) using the Bhattacharya method across age groups from 2 months to 18 years, categorized by age and gender. Moreover, we have added upper and lower limits for the age group of newborns to 2 months. These values were determined using the non-parametric method, with the 2.5th and 97.5th percentiles specified. This method was chosen as a baseline for establishing reference values from data exhibiting a non-Gaussian distribution. Specifically, in the newborn to 2-month age group, both male and female data showed a non-Gaussian distribution with a significance level of P-value < 0.05. Despite our efforts to normalize the distribution using statistical methods such as Tukey's test and Box-Cox transformation, the data remained non-Gaussian.

Age	Sex	n	LRL*	URL*
0-60 d***	F	1851	0.34	1.04
	M	2578	0.34	1.06
2-12 m	F	1334	0.15	0.34
	M	1829	0.15	0.35

1-<3 y	F	1811	0.18	0.40
	M	2311	0.18	0.40
3-<5 y	F	1280	0.22	0.46
	M	1603	0.21	0.48
5-<7 y	F	1117	0.24	0.52
	M	1473	0.26	0.54
7-<9 y	F	1113	0.29	0.57
	M	1285	0.26	0.55
9-<11 y	F	1076	0.29	0.60
	M	1235	0.31	0.61
11-<13 y	F	931	0.32	0.64
	M	1155	0.34	0.71
13-<15 y	F	790	0.32	0.75
	M	943	0.36	0.82
15-18 y	F	488	0.37	0.77
	M	443	0.50	1.00

URL = Upper Reference Limits, LRL = Lower Reference Limits 0-60 d*** = non – parametric method

5. Page 15, lines 272-275

You could make a more confident claim if you used inferential statistics to evaluate this observation

We appreciate your valuable suggestion to use inferential statistics for strengthening our claims. In the process of establishing reference values for serum creatinine in this study, the research team systematically followed the CLSI EP28 A3C guidelines at every step, employing appropriate statistical methods. Given that this study focused on the pediatric population spanning from birth to 18 years old, where physiological characteristics evolve continuously, it's crucial to highlight the significance of gender differences beyond just age. For instance, the

study examined whether there are variations in upper reference limits (URLs) between males and females within each age group. The URL is a significant indicator of creatinine's importance in assessing kidney function. For example, in the 15-18 years age group, a notable difference was found between males (1.00 mg/dL) and females (0.77 mg/dL) in URL values. To ensure appropriate and specific reference values for each subgroup, it's important to highlight such differences. The demonstration of such differences in URLs, being the upper threshold of the reference values, is crucial. From the examples, differences in reference values were observed up to 26%. While statistical methods like Independent-samples t-test, Mann-Whitney U Test measure trends towards the central tendency of data (mean, median), they may not capture the differences in URL points we are interested in. Therefore, comparing URLs using percentage differences is considered statistically sufficient and appropriate for assessing differences in URL values between males and females within each age group.

Page 16, lines 296-299

6. Could it also be due to dietary differences? e.g. which of these countries have a higher meat consumption per capita?

Thank you for your insightful comment regarding the potential influence of dietary differences, particularly meat consumption, on serum creatinine levels in our study population.

We greatly appreciate your suggestion and have conducted an analysis of meat consumption per capita, referencing data from 2020. Our findings indicate significant variance among countries, with Thailand exhibiting relatively lower meat consumption compared to countries with higher GDPs. This observation is crucial, as dietary habits, especially protein intake from meat, can impact serum creatinine levels due to their association with muscle metabolism.

In our revised manuscript, we have incorporated this analysis to highlight the importance of considering dietary habits alongside economic factors when interpreting serum creatinine levels, particularly in pediatric populations across different countries. Recognizing that countries with higher meat consumption may reflect larger muscle mass rather than impaired renal function, this contextualization enhances the applicability of the reference ranges we have established in clinical settings.

Thank you once again for your valuable input, which has enriched the discussion in our manuscript. The revised sentence has been added to the manuscript on **Page 16, Lines 292-295.**

Reviewer 2

1.Establishing Age and Gender-Specific Serum Creatinine Reference Ranges for Thai Pediatric Population

Response 1: Thank you for your detailed and insightful review of our manuscript titled "Establishing Age and Gender-Specific Serum Creatinine Reference Ranges for Thai Pediatric Population." We appreciate your thorough assessment of our study and your valuable feedback.

2. Introduction

Creatinine, a widely used renal biomarker, aids in diagnosing and monitoring acute and chronic kidney diseases. However, factors like muscle mass, sex, ethnicity, and age influence its levels. This study addresses this crucial gap by establishing reference intervals for Thai children and adolescents (under 18 years).

The subject is clearly explained with a strong background highlighting the study's significance. The research aim is also explicitly stated.

Response 2: We are pleased to hear that you found the introduction clear and the background of the study well-explained. We agree that the factors influencing serum creatinine levels, particularly in pediatric populations, are complex and multifaceted. Our aim was indeed to address this crucial gap in the literature by establishing reference intervals tailored specifically to Thai children and adolescents.

3. Methodology

Retrospective data from the Queen Sirikit National Institute of Child Health (January 1, 2017, to December 31, 2021) was used. The analysis included 29,224 entries, representing the first creatinine test per individual, avoiding data duplication issues.

The sample size, with sufficient data points, is appropriate for reference interval studies. Serum creatinine analysis employed the well-established enzymatic colorimetric method on the COBAS INTEGRA 400/400 PLUS analyzer. Both internal and external quality control data were presented, ensuring the reliability of reference value interpretation.

Including data from other analyzers or test sites within Thailand could strengthen the generalizability of the reference intervals, as they will be applied across various platforms. Data from diverse sources helps eliminate errors and biases inherent to single sites or instruments.

Response 3: We are grateful for your acknowledgment of the appropriateness of our sample size and the rigorous methodology employed in our study. Your suggestion to include data from other analyzers or test sites within Thailand is well-taken and could indeed enhance the generalizability of our reference intervals. We will consider incorporating data from additional sources in future studies to further strengthen the reliability and applicability of our findings. The revised sentence has been added to the manuscript on **Page 20, Lines 372-378.**

4. Results/Analysis

Results are clearly presented in easy-to-understand tables and charts, categorized by age group and gender for improved analysis. Graphs and figures are well-labeled and comprehensible.

Outliers were rightfully excluded, and non-Gaussian data was normalized using standard statistical tools.

However, the very large SD/CV for male 5–7-year olds' serum creatinine results require explanation. It might bias the reference range for that age group.

Response:

We apologize for any confusion or concern caused by the large standard deviation/coefficient of variation observed in the serum creatinine results for male 5–7-year-olds. We appreciate your diligence in identifying this discrepancy and bringing it to our attention.

Upon further investigation, we acknowledge that such a significant deviation could potentially bias the reference range for that specific age group. To address this issue, we have thoroughly scrutinized our data and made necessary corrections to ensure the accuracy and reliability of our findings.

In our revised manuscript, which now includes **Supplementary Information (S1 Table)**, we have amended the standard deviation and coefficient of variation values for males aged 5 - <7 years. Specifically, **we have corrected the standard deviation from 0.38 to 0.08 and the coefficient of variation from 97.44 to 20.51**.

We are committed to transparency and accuracy in our research, and we thank you for your valuable feedback, which has contributed to improving the quality of our study.

5. General Remarks

This well-written paper clearly presents the background, aim, methodology, and results. The researchers acknowledge the study's limitations, and further improvement in accuracy and scope using different statistical tools is encouraged

Response 5: We are grateful for your overall positive assessment of our paper and your encouragement for further improvement in accuracy and scope using different statistical tools. We will carefully consider your suggestions and explore opportunities to enhance our study using alternative statistical approaches to ensure the robustness and reliability of our results.

Once again, we sincerely appreciate your constructive feedback and your commitment to advancing scientific research in this field.
