# Supplementary material for: Establishing age and gender-specific serum creatinine reference ranges for Thai pediatric population
Source: PLoS One. 2024 Mar 12;19(3):e0300369. doi: 10.1371/journal.pone.0300369 (PMC10931469; doi:10.1371/journal.pone.0300369)
Supplement: Attachment — Submitted filename: Review- Establishing Age and Gender.docx [file pone.0300369.s003.docx]

**Establishing Age and Gender-Specific Serum Creatinine Reference Ranges for Thai Pediatric Population**

**Introduction**

Creatinine, a widely used renal biomarker, aids in diagnosing and monitoring acute and chronic kidney diseases. However, factors like muscle mass, sex, ethnicity, and age influence its levels. This study addresses this crucial gap by establishing reference intervals for Thai children and adolescents (under 18 years).

**The subject is clearly explained with a strong background highlighting the study's significance. The research aim is also explicitly stated.**

**Methodology**

Retrospective data from the Queen Sirikit National Institute of Child Health (January 1, 2017, to December 31, 2021) was used. The analysis included 29,224 entries, representing the first creatinine test per individual, avoiding data duplication issues.

**The sample size, with sufficient data points, is appropriate for reference interval studies. Serum creatinine analysis employed the well-established enzymatic colorimetric method on the COBAS INTEGRA 400/400 PLUS analyzer. Both internal and external quality control data were presented, ensuring the reliability of reference value interpretation.**

**Including data from other analyzers or test sites within Thailand could strengthen the generalizability of the reference intervals, as they will be applied across various platforms. Data from diverse sources helps eliminate errors and biases inherent to single sites or instruments.**

**Results/Analysis**

**Results are clearly presented in easy-to-understand tables and charts, categorized by age group and gender for improved analysis. Graphs and figures are well-labeled and comprehensible.**

**Outliers were rightfully excluded, and non-Gaussian data was normalized using standard statistical tools.**

**However, the very large SD/CV for male 5-7 year olds' serum creatinine results requires explanation. It could be a typographical error, but if not, it might bias the reference range for that age group.**

The WHO-recommended Bhattacharya method established reference values, and the upper and lower limits were compared to three similar studies in this age population.

**General Remarks**

This well-written paper clearly presents the background, aim, methodology, and results. The researchers acknowledge the study's limitations, and further improvement in accuracy and scope using different statistical tools is encouraged.
